# Recovery through creativity: a study into lived experiences of participants in Dutch arts-based recovery initiatives

**DOI:** 10.3389/fpubh.2026.1764635

**Published:** 2026-04-15

**Authors:** Jenny Boumans, Arko Oderwald, Hans Kroon

**Affiliations:** 1 Trimbos-Institute, Utrecht, Netherlands; 2 Department of General Practice, Amsterdam University Medical Center, VUmc, Amsterdam, Netherlands; 3 Tranzo Scientific Center for Care and Welfare, Department of Social and Behavioral Sciences, Tilburg University, Tilburg, Netherlands

**Keywords:** arts, creativity, lived experiences, mental illness, qualitative research, recovery, RTA

## Abstract

**Introduction:**

It has been suggested that participatory arts-based activities inherently align with recovery-oriented way of working and may aid personal recovery in people with mental illness. However, underlying mechanisms need to be further clarified. The aim of this study is to contribute to more understanding on recovery through creativity by examining lived experiences in the context of arts-based recovery initiatives.

**Methods:**

In this qualitative, interpretive study, 26 participants from Dutch arts-based recovery initiatives were purposefully selected. Unstructured interviews were conducted and transcribed. The data were analyzed using reflexive thematic analysis.

**Findings:**

Eight themes describe participants' lived experiences of personal recovery through creativity in the context of participatory arts initiatives. We grouped these into four metathemes that represent the underlying mechanisms of recovery through creativity: “Engaging in a conscious and constructive activity,” “Transforming and transcending painful experiences,” “Belonging in a creative community,” and “Steps toward emancipation.” A fifth metatheme, “Opposing forces,” describes the processes that hinder a favorable relationship between creativity and personal recovery.

**Discussion:**

The research confirms that participatory arts initiatives align with the recovery philosophy, with both contextual and arts-related aspects playing a role in the perceived benefits of participating in such an initiative. The experience of beauty and a sense of wholeness as a counterbalance to experiences of psychological suffering offers a promising avenue for further research, as well as research leading to a better understanding of how opposing forces in the relationship between creativity and recovery can be (self-)managed.

## Introduction

There is increasing interest in the potential of artistic activities in improving health and wellbeing, and a growing consensus that they should be an integral part of modern healthcare, including mental healthcare (1–6). The idea that art can contribute to positive health effects is, however, not new. For almost a century, creative and artistic practices have been used as part of treatment for people with mental illness, mainly in the context of inpatient psychiatric care (7). This approach has traditionally been dominated by a clinical, therapeutic perspective. Recently, there has been an expanding academic interest in the effects of community-based participatory arts activities on personal recovery from mental illness (8–16), which is in line with deinstitutionalization movements and the emergence of the Recovery paradigm.

The concept of personal recovery is a response to the narrow interpretation of recovery from a clinical paradigm that pays little attention to the loss of identity, purpose and self-esteem associated with mental illness (17). Personal recovery can be described as a deeply personal, unique journey toward a satisfying, meaningful and rewarding life, characterized by discovering a new sense of identity and purpose, even with the limitations caused by illness (18, 19). A recovery-oriented approach calls for equitable and reciprocal relationships between service users, staff, and career stakeholders, and a focus on personally defined recovery (20). Participatory arts-based activities are shown to naturally complement recovery-oriented approaches, fostering key elements such as empowerment, connection, agency, and hope (21–23), which might aid recovery in other ways than, for example, regular therapies do (24).

Participatory art activities differ from more traditional forms of art therapy, which requires therapists with specialist training, in that they are focused on the process: actually “doing” and experiencing the creation of art (22, 25). In other words: art is primarily created for the sake of the art. Many benefits of participatory arts activities in people with mental illness have been described, and include improvements in empowerment, social inclusion, confidence, motivation, self-awareness, wellbeing, as well as overall quality of life and mental health (8, 10, 11, 15, 26–31). As most of these benefits have yet to be validated in controlled settings, they should be interpreted with caution, leaving room for critical reflection—or, as Clift et al. (1) suggest, “balancing optimism with assessments of uncertainty.”

Much also remains unknown about the underlying mechanisms through which creativity can contribute to personal recovery. It is suggested that arts initiatives offer a safe and empowering community-based environment—one that is non-judgmental, structured but flexible, focused on potential rather than limitations, and conducive to collaboration and co-production. Such settings foster creative freedom, equality, choice and meaningful connections and provide space for sharing and telling personal stories (12, 32–35), which may facilitate the sense of connectedness and belonging and offers the opportunity to develop skills and redefine one's own identity (8, 16, 36–39). This, in turn, is assumed to promote recovery. Mechanisms inherent to the creative process itself also appear to play a role, These include, for example, the experience of artistic flow as a distraction from negative thoughts, the expression and transformation of difficult experiences and emotions in art, and the narrative potential of art to challenge current perceptions of mental illness (16, 30, 40).

Further research is needed to deepen our understanding of how creativity can contribute to recovery. Most studies addressing this question are based on small sample sizes and tend to focus on the characteristics and perceived benefits of specific arts-based programs or interventions. In the present study we are interested in the creative process itself as well as the context in which creativity unfolds. To gain deeper insight into this issue, the study explores the lived experiences of a large and diverse group of artists with lived experience of mental illness participating in Dutch arts-based recovery initiatives. This approach aims to give voice to people who have personal experience with processes of recovery and offers the opportunity to study the coherence of different aspects of recovery and creativity in the context of the person's life. The study is informed by the following research questions:

RQ 1: How do participants in arts-based recovery initiatives experience the relationship between creativity and personal recovery?
RQ 2: What can these experiences tell us about the potential mechanisms of action of arts-based recovery initiatives?

## Methodology

### Design

A qualitative design, pragmatically guided by Reflexive Thematic Analysis (RTA) (41–43). Underlying theoretical assumptions included a constructionist, experiential and inductive approach to research, which is the most appropriate theoretical stance for the purpose of the study as it gives space to personal experiences of participants. We used insights from hermeneutic-phenomenological thought (44) as an inspiration for our methodological approach. Hermeneutic-phenomenological thinking assumes that there is no “single universal truth” about phenomena. It follows a subjectivist/interpretivist ontology that focuses on the way in which individuals experience, describe, interpret and understand a particular phenomenon—in this case recovery through creativity—and is characterized by a continuous, non-linear, circular process of interpretation (45). In this paradigm, research can never be considered value-free. Instead, it assumes a value-laden axiology. The researcher is regarded as an integral part of the analysis, as a tool for understanding. Their task is not to arrive at a definitive answer, but a plausible, contextualized, and credible account of what an experience means to a particular group of participants. The researcher's active role in knowledge production and the analytical process is thus not avoided but acknowledged and embraced (46), which is also a core principle in RTA (47). The analysis in this paper is part of a larger study into lived experiences with creativity and mental illness, conducted by the first author.

### Recruitment

For this study, we recruited participants of Dutch arts-oriented recovery initiatives for/by persons with experience of mental illness. The scope of different disciplines in, and approaches to, art practices in the Netherlands is broad; however, within this study we limited ourselves to visual and performance art. Two educational art/recovery centers, four arts-based daytime services, two Living Museums and three art groups/collectives of artists with experience with mental illness were involved in the study. Of these 11 organizations, six focus on visual art-making and five on performing arts (music, theater). Characteristic of these organizations is that they eschew a therapeutic paradigm: their emphasis is not on working through personal issues to achieve psychological change, as it is in many forms of creative therapy. Instead, the organizations are art-oriented, that is, making art is the primary goal, while the potential of collaborative art-making to produce positive change in people's health and lives is secondary. Although some organizations are linked to mental health organizations and occasionally employ mental health professionals such as art therapists, leadership tends to be by professional artists. All organizations accept participants regardless of their initial level of artistic ability.

### Approaching and informing potential participants

In all organizations a central contact person (often a project leader or the founder of the organization) mediated in the recruitment. After initial contact, and if the investigator determined that the person met the inclusion criteria, the potential participant received an official letter about the study, which included GDPR guidelines on confidentiality and data processing as well as a consent form, and an interview appointment was scheduled. It was emphasized that participants could withdraw from the study at any time.

### In- and exclusion criteria

Inclusion criteria were as follows: over 18 years old; current or past participation experience in a Dutch arts-based recovery initiative for at least one consecutive year; self-reported mental illness with clear, long-lasting effects on everyday life; Dutch- or English- speaking and living in the Netherlands. No essential exclusion standards were used. For example, florid signs of conditions like depression or psychosis were not a criterion for exclusion. Since the aim was not to investigate the interventions/programs themselves but rather people's experiences with creativity and recovery, we did not distinguish between types of arts activities, frequency of participation, etc.

### Participants and sample size

We recruited participants until theoretical saturation was reached. We are confident that the final sample size of 26 participants is sufficient to achieve a high level of completeness, comprehensiveness, and philosophical consistency (48), and made it possible to look for potential similarities (shared experiences) and differences (uniqueness).

### Data collection

The first author conducted a single, unstructured, in-depth interview with each participant. The interviews lasted an average of 63 min (with a range of 40–98 min). All interviews were tape recorded, transcribed verbatim by the first author, and pseudonymized. The researcher took field notes about her impressions both during and after the interview, as well as during transcription, and actively reflected on her own assumptions and interpretations during the process of data collection. The majority of interviews were conducted remotely, by means of a video call, except for five face-to-face interviews at the location of the arts initiative, and one by phone. Although a live interview allowed more space for the researcher to see and experience the person's artistic work, there were no noticeable differences in the data gathered live vs. remotely. Both forms of conversation led to good, in-depth conversations about experiences. After the interview, a short questionnaire with general questions (both demographical and related to art discipline and mental illness experience) was administered.

### The interview

The nature of the interview was “open,” which means that the participant's experiences were central, and the researcher primarily listened and interfered minimally (49). The goal of this interviewing method is to gain insight into personal meanings and experiences (not guided by any theoretical or empirical frame of the researcher) related to the topics of creativity and recovery within the context of the participants' own life (story). This also meant that the topic list was kept fairly simple. It consisted of a first question (that invited participants to start talking) and a number of main topics, which were not prescriptive and left plenty of space. Each interview began with a detailed explanation from the researcher about the study's background and about her own relationship to the research topic. The participant was then invited to share something about the art discipline they are involved in, how the interest in this art discipline arose and how they came to be involved with the art initiative. These questions seemed to be a good starting point to begin discussing creativity, mental illness and recovery. Participants were in charge of narrating their experiences; follow-up questions from the researcher always matched the story of the participant. The researcher helped to make the story coherent with active and deep listening, and by picking up things that seemed to have meaning for the interviewee. Co-constitution techniques were used to validate the participants' meanings and the researcher's understanding (50).

### Ethical issues

All procedures were in accordance with both the ethical standards of the Trimbos Institute and the 1964 Helsinki Declaration and its later amendments or comparable ethical standards. Written informed consent was obtained from participants. The option to withdraw was made clear. To safeguard the privacy of the participants, personal information was omitted from the transcripts. After transcribing, audio files were deleted. This non-invasive, qualitative study was judged exempt from a review by an external review board. Specific recommendations of the ethics committee of the Trimbos Institute to optimize procedures of secure processing of personal data, the provision of full and understandable (legal) information on study participation and assessment of the burden of the interview, were adopted. (Ethical procedure Registration Number: 3028631/25-05-2020).

### Data analysis

The aim of the data analysis was to gain insight into the experiences of people participating in an arts-based recovery initiative and in the meaning and meaningfulness of creativity within recovery processes. We applied Reflexive Thematic Analysis (RTA) to identify and analyze patterns and themes across the data by organizing codes around a relative core commonality, or “central organizing concept,” that the researcher interprets from the data (41–43). The actual analysis included a six-phase process of familiarization, initial (latent) coding, theme generation, reviewing and revising, working toward a thematic framework, and writing up findings (51). To identify themes we applied principles from hermeneutic-phenomenological methodology such as “dwelling with the data” (45), which amounts to a circular process of reading and re-reading the parts and the whole of the transcriptions, interpretation, distilling (increasingly nuanced) themes and merging similarities into common themes and shared meanings. By adopting a constructionist epistemology, the researcher acknowledges the importance of recurrence, but appreciates meaning and meaningfulness as the central criteria in the coding process (47). The process of coding was aided by using MAXQDA software for qualitative analysis, and focused on latent meanings and underlying assumptions, ideas, or ideologies.

### Reflexivity and rigor

In qualitative research—and particularly in hermeneutics—it is assumed that being situated within or connected to the phenomenon of interest can significantly enhance the researcher's ability to discover meaning (45). While her involvement cannot be seen as an insider perspective, the lead author did bring a variety of prior experiences and knowledge on the study's main topics. The lead author, who is trained in health sciences, had over a decade of experience in applied mental health research, with a particular focus on recovery and recovery-oriented practice. She conducted numerous interviews with people with mental illness, including about what recovery means outside of a medical model. The content of these conversations also touched on personal experiences surrounding (the search for) meaning and eudaimonic wellbeing. Furthermore, she had been active in the arts as an amateur musician since childhood and regularly experienced in herself and others that creativity can have something to do with mental wellbeing. All these professional and personal experiences shaped her prior understanding of the phenomena under study and led her to explore how these concepts might intersect and how their potential relationship might be understood.

However, the benefit of being connected to the research subjects rests on critically considering the influence of the researcher's own position and assumptions (46, 52). To document and explore her thoughts and preconceptions about creativity, mental illness, and recovery, the principal investigator kept a reflective journal throughout the research process. This journal proved particularly valuable during the data analysis, as it helped her explore how her own perceptions related to those of the participants, nuance her initial interpretations where possible, and approach the participants' stories with greater openness. The addition of two researchers with different academic backgrounds (medical humanities, social sciences) to the research team broadened the interpretation of the data and helped to critically question personal and societal understandings of art and recovery, thereby increasing the scientific rigor of the study.

The authors further strengthened the study's validity and reliability by providing a robust and transparent account of the research setting, participants, data collection methods, and analytic procedures, supported by rich, thick descriptions of participants' interpretations (53). They demonstrated how the findings relate to the broader literature and used verbatim quotes from participants to support the presented results and illustrate both shared and divergent experiences (54). In presenting the findings, some excerpts retain the first author's (“JB”) questions or responses to clarify the flow of the dialogue for the reader.

### Characteristics of study participants

Study participants (see Table 1) ranged from age 28–69 years (mean 52), and included 14 women (54%), and 12 men (46%). Of the study participants, 54% were involved in visual arts (painting, drawing, digital art, combined techniques), and 46% in performing arts (music, theater). In terms of education, 50% had completed vocational training, 35% finished higher education, while 15% had only finished high school. Before attending the art recovery initiative, 84% were already involved in their art discipline: 19% had learned the art discipline by themselves without lessons, 50% had learned their discipline in the amateur art education circuit and 15% had received some formal art or art-related training (one person started art academy but didn't finish it, one person finished a creative study on vocational level, 1 person finished a private art-related education program, and one person finished an online course to become a creative therapist). The other participants (16%) started being involved in the art discipline only when joining the art initiative. A quarter of the participants (26%) earned (some) money with art.

**Table 1:** Characteristics of study participants.

	Pseudonym	Age (years)	Gender	Art discipline	Art practice	Self reported main diagnosis
1	Ronald	66	M	Music	Art education center	Psychosis
2	Brian	33	M	Music	Art education center	Psychosis
3	Edward	51	M	Music	Art education center	Bipolar disorder
4	Jacob	67	M	Visual arts	Living museum	Anxiety/compulsive disorder
5	Victoria	54	F	Visual arts	Living museum	Depression
6	Henry	55	M	Visual arts	Art daytime services	Bipolar disorder
7	Frank	48	M	Visual arts	Art daytime services	Bipolar disorder
8	Hannah	55	F	Visual arts	Art daytime services	PTSD
9	Mark	28	M	Visual arts	Living museum	Personality disorder
10	Patricia	42	F	Visual arts	Living museum	Depression
11	James	42	M	Visual arts	Living museum	PTSD
12	Linda	52	F	Theater	Art collective	Personality disorder
13	Richard	63	M	Theater	Art collective	Depression
14	Daniel	69	M	Visual arts	Art education center	Depression
15	Elizabeth	61	F	Visual arts	Art education center	Depression
16	Olivia	33	F	Theater	Art collective	Personality disorder
17	Gary	58	M	Visual arts	Art daytime services	Psychosis
18	Christine	64	F	Visual arts	Art daytime services	Psychosis
19	Julie	28	F	Theater	Art collective	Eating disorder
20	Judy	55	F	Music	Art education center	Psychosis
21	Evelyn	32	F	Theater	Art collective	Depression
22	Margaret	63	F	Theater	Art collective	PTSD
23	Harold	52	M	Visual arts	Art collective	Anxiety/compulsive disorder
24	Virginia	63	F	Visual arts	Art education center	Depression
25	Amanda	61	F	Visual arts	Art education center	Personality disorder
26	Janet	49	F	Theater	Art collective	PTSD

Self-reported main mental illness experience was psychosis-related (19%), mood- related (34%), anxiety/compulsivity-related, including PTSD (23%) and “other” mental illnesses (23%), including eating disorders, autism, dissociative disorder and ADHD. Self-reported comorbidity was high: 65% reported a second mental illness, 30% a third, 15% a fourth. At the time of the interview, 76% were receiving mental health care. Another 12% had received mental health care in the past but were, at the time of the interview, supervised and treated by their general practitioner; while 12% no longer received any kind of mental health care.

## Findings

**Figure 1:**
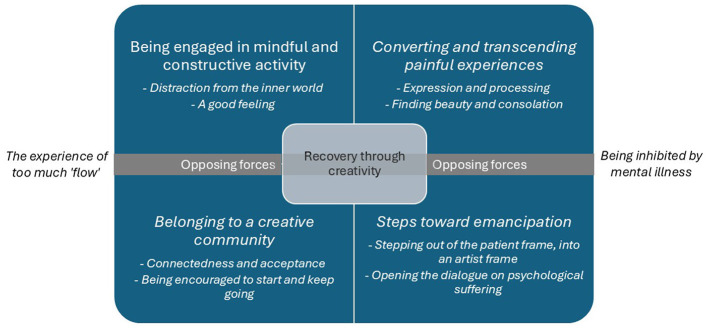
Thematic framework, inductively obtained through analysis of experiences.

The most evident, overarching pattern we identified in the interviewees' stories was that the meaning of creativity for recovery can be understood as an interplay of creativity-related and collective meanings. In order to gain a better understanding of how these different meanings take shape, we focused on the underlying lived experiences. We identified eight themes that describe these experiences and clustered them in four meta-themes, of which two relate to the beneficial aspects of the creative process itself and two relate to the collective opportunities offered by the art initiative. The four meta-themes are: “being engaged in mindful and constructive activity”; “converting and transcending painful experiences”; “belonging to a creative community”; and “steps toward emancipation.” The data show, however, that tensions can emerge that hinder the beneficial potential of creativity for personal recovery. We described these tensions under a fifth meta-theme: “Opposing forces.” The five (meta)themes are shown in Figure 1 and further explained below.

### Being engaged in mindful and constructive activity

*Distraction from the inner world:* Participants, firstly, experience engaging in creative activities as a way to get into a positive flow or state of mindfulness that distracts them from compulsively wandering thoughts and worries and brings peace of mind. Characteristic of this experience in the descriptions is the sense of timelessness, the ability to focus on the here and now because “you really have no choice but to go along with the process.” As Jacob explains, when he is drawing, he focusses completely on drawing, so he doesn't have to think about “all the other 258 things on his mind”. Hannah similarly phrases it as a distraction; from herself and from the world. Almost all participants share this experience of distraction and flow and value it for its calming and stress-reducing potential.

Virginia: I prefer to be a little without words, and when I am making art it is also without thinking. Do you understand? It is rest for your thoughts. Because in my head it's all rattling around. Well, when I'm drawing, I'm actually waiting for what's to come. And then I sit and follow my own hand or that brush and then, “Ooh, beautiful!” Do you understand? The best thing about it is that the thoughts become silent. And then I no longer need that OCD to calm me down. It is as if art says “Let it go, let it go.”

In sharing experiences about how creativity leads to distraction, all participants emphasize the intuitive nature of the creative process. Just “being” with the process of creating, and being surprised by what arises, is very important. Visual artists add to this the value of the tactile quality of the process of making art: experiencing how the material you work with *feels*, and the focus on shapes and colors, which ensures that you have nothing else on your mind. For musicians, it is the vibrations produced by sounds and melodies and the ability to sail along on the different dimensions, moods and emotions that are contained therein. For participants doing theater, the feeling of surrender when playing a scene or taking on a certain role, and the focus that is needed for this, ensures that one's own thoughts become quieter.

Richard: I think I end up in a kind of daze, of yes in the moment, now it has to happen, and surrendering myself completely to the play and to the performance.

*A good feeling:* Engaging in the creative process not only quiets the mind, but also produces affective effects, which often linger beyond the creative process, and are perpetuated with more regular art practice. Participants describe how making art lifts their mood and gives them (at least when things go well) a sense of success and satisfaction, which can help build self-esteem. As Henry explains, it makes him feel good—really good. He feels competent at something; many things don't work out, but painting does. Patricia adds that the creative process has helped her feel, and accept, herself more. Her self-image has improved enormously because of it.

Participants emphasize the difference between making art, which is a constructive activity, and other, more passive, activities such as watching TV or doing household chores. Making art requires a specific effort and commitment, and also offers the opportunity to share something (positive) of yourself with others. It is precisely because of these kinds of qualities that it can gradually counterbalance deep-seated and persistent feelings of shame, self-disappointment and worry.

Hannah: I was very disappointed in myself, I was very disappointed in life. My self-esteem was very low, I didn't know where I was going, I felt very low on energy. And then [when starting to make art] everything came up you know? So the creative process was in a way healing for me. And I think it still is, in a way it is therapy I think without noticing.

For some participants making art, through its constructive quality, has become their most important coping mechanism in dealing with symptoms of mental illness. These participants explain that when they are under-involved in creative processes, they experience an increase in symptoms, a decrease in self-confidence and they become more vulnerable to relapse. Conversely, they use creativity the moment they start to feel worse, and through the creative process they manage to return to calmer waters.

Edward: Doing things of which you know: that makes me feel good, that makes me feel a bit better, that's what your recovery is about. So those are things I'm going to do a lot [when not doing well]. Drawing, painting, and making music are my way out of depression.

### Converting and transcending painful experiences

*Expression and processing:* This theme revolves around the rather unique ability that art offers to put feelings, experiences and thoughts into the thing you make. Participants describe that the moment something from their inner world ends up in their art it is transformed into something else, something that is tangible and can be viewed from a distance. That makes it possible to experience it, interact with it and move through it—but without the fear of getting lost in it.

Olivia: Performing is sometimes difficult, especially when what you're performing is so close to your own life. But when I'm performing, I don't experience that. I'm preoccupied with where to stand, what words to say, where to look, maintaining an open posture. All those technical things come up. And then I work through that painful content, but from a distance. It contributes to the healing because it creates a kind of space for a part of myself that's difficult to accept, that I always carry with me... It's part of the processing, or something.

Participants experience the conversion of feelings, thoughts and experiences in art as liberating and redemptive as they often find it difficult to process these in other ways, especially when it concerns confusing or heavy thoughts. As Ronald shares, it feels like a liberation from a shackled existence. In the creative process, he is able to transform depressive feelings and find a ‘solution' to his issues.

Participants emphasize that it is important to give meaning and space to painful experiences in their art, not because they then become less difficult, or because they are then immediately cured of them, but because they are part of their life story and should not be hidden. In the form of art, these parts are allowed to exist, albeit in a more abstract form, which helps them to come to terms with it.

James: I'm processing what I've been through, and I think that's really important. It's a way to come to terms with it. I see it as a form of recovery. Sometimes it's difficult to talk about your problem, but you can also process it in a poem, or you can tell your story on canvas. I think it's simply a form of processing. That's how I see my art: as a form of processing.

Furthermore, many participants describe how art gives the opportunity to not only to consolidate experiences for themselves but also to transmit them to others, which leads both to a meaningful shared artistic experience and shared understanding with, for example, loved ones, of the things that are contained in the piece of art.

Julie: It was very important to me, so others could see it too. Because i felt misunderstood during that period. That performance felt like a kind of processing. People close to me, like my mother, naturally came to see it too. And yes, I would never have said, “This is actually about me,” but in the meantime, I'm showing myself, what I really want to tell them, what it's like for me. I'm showing it to them without saying it's about me. And it also led to a better connection with my mother; yes, I have a better connection with her because of it. I just wanted her to understand me.

For some participants, being able to express experiences in artistic ways also quite literally helps them to prevent more destructive ways of expressing it. As Linda explains, expressing her feelings through art, and processing and communicating her emotions that way, keeps her emotions from coming out - in her words - all “borderliny.”

Incidentally, it is important to add that for most participants, processing and converting painful experiences is not the *primary* goal of making art, but rather a consequence. It can be a largely unconscious, indirect process, in which the artist only notices during the art-making process or afterwards that the artwork contains experiences that are easier to accept and transfer to others because of their new form. The process is also not limited to matters that are difficult or stressful. Other experiences, feelings and ideas can also be given a place in art, and therefore get the right to exist.

Mark: Most of it certainly is part of my inner world. I am not much of an emotional talker at all, I also find it very difficult to talk about feelings. But in art it comes out anyway. I can put it into a kind of mold that shows how complicated I think it is. This is kind of poetic. But truth. But it is not only stressful things. Sometimes I just feel like: wow that is interesting or weird. Not that it really destroys me, but that I think: I have to represent this in a certain way. Because I find that interesting too.

*Finding beauty and consolation:* Yet another dimension to the process of converting experiences that contributes to the wholesome potential of art making is the fact that - making art can lead to an experience of beauty and wholeness. Participants describe that such an experience has the capability to counterbalance (some of) the crudeness and “ugliness” of experiences with mental illnesses. Some participants specifically look for experiences of beauty. Virginia, for example, who shares that, in contrast to her mental illness, she always strives for harmony or peace in her work, or as she puts it: “something sweet through form or color,” “an image that radiates a kind of melody.” The elicitation of an experience of beauty, however, most of the time happens unexpectedly, and may even arise when very difficult themes have been or are being consolidated in the work of art. According to participants, this is because personal, singular experiences and stories, including the “ugly,” are placed in a larger, more universal context through art, and as such can contain a form of beauty.

Victoria: Suffering in itself is only ugly, but a *story* about suffering, hidden in a work of art, can be very beautiful. Beauty can be in so many things, but it is especially in the ugly. We normally don't see that. Yes, I like that aspect of art. I like the story behind the surface, the frayed edges of existence, empty buildings. It doesn't need to be smoothed out for me.

The experience of recognizing a form of beauty within suffering, through art, is described by several participants as a form of consolation, which is more than just coming to terms with it. Rather, it is described as a very profound experience of meaning, which is confrontational and comforting at the same time, and as such forms an opportunity to be a whole person again, despite and because of suffering.

Olivia: It was so beautiful, I sometimes really had to swallow tears because I really saw myself in it and I felt really disgusted, but at the same time it was also beautiful and a recognition and a kind of consolation that I felt. Very special.

Edward: I think a lot depends on the experience of beauty for me in the end. This makes me emotional, but it … it makes me feel whole inside … euh … it is consolation … yes … it is comforting absolutely. I find beauty comforting. I have learned to like my emotions too. Maybe I should say that too. I'm a very emotional person and when I'm sad I can even enjoy the fact that I'm crying or something. JB: So you also experience beauty in your own suffering? Edward: Yes. Yes. Yes. Sure.

### Belonging to a creative community

*Connectedness and acceptance:* Where the previous themes dealt with aspects related to the creative process itself, the following themes deal with aspects related to the context offered by the art-oriented recovery initiatives. For participants, the art initiatives they attend represent a physical place ánd social community where they meet like-minded people and where people are seen and accepted for all they are and have, including their difficulties. Participating in such an initiative, for some a completely new experience, was described as very stimulating and affirming.

Victoria: The founder said, “How about this table?” He takes a thick marker and writes my name in large letters on the wall above that table. And I really stood there crying. Then he handed me over to one of the other artists there for a tour. And it felt so much like coming home, such a relief. I felt so unconditionally at home. And from that moment on I felt it. Yes, you don't have to explain yourself here, you won't be judged. If it's not my day, that's accepted. I'm not that talkative, don't necessarily have to join the conversation, but the feeling that you are connected to each other… Connection with like-minded people, that's what I was looking for.

The sense of connection and community that arises in such art initiatives is, according to participants, strongly related to the fact that participants have similar (mental illness) experiences, which ensures a lot of mutual understanding and acceptance, without having to actually touch on the topic. Patricia's experience is illustrative for this: at the art initiative she attends, one can simply walk in with a sad face, and everyone will understand.

Even more meaningful, however, seems to be the shared passion for art. It is liberating to meet others who have the same interest. For Mark, who came from a period in which he was living in a very small bubble, entering the art initiative meant realizing for the first time that there are many other people who create art in the same way he does. Participants value the genuine interest shown in each other's work and the collective willingness to help each other grow personally and artistically, which contrasts with other environments, where pressure and expectations often play a role.

Hannah: You can really grow here in what you do. And that happens here because the interaction is different from what you're used to; it's a different kind of interaction. JB: Yes? What's different about it then? Hannah: There are no expectations of you. In every other situation, there are expectations you have to meet. And not here. Normally, there's a lot of judgment and pressure in society. Here, there's no pressure at all. You can just be who you want to be. And when people are themselves, you can become stronger. This place is so special. The interaction between the people and the people who run this place—they're special people, you know, they give people space.

Daniel: I did a course at a regular art school: modern art, six evenings, and then you come across a group of people where you suddenly have the feeling: there is competition, who can tell their story the best, who is the smartest. I had that feeling then and that is not the case here at all. Everyone can be themselves and you have a lot of interest in each other. Yes, that is just a very pleasant environment.

Participants emphasize that the shared passion for art and equality between participants lays the foundation for the formation of attachments, which over time can even function as a safety net and/or lead to close friendships. As a result, participating in an art initiative contributes to getting out of a situation of social isolation and loneliness and moving toward a situation of (more) connection and belonging. It is, as it were, an antidote to loneliness, and therefore an opportunity for recovery. As Jacob puts it, if a person doesn't communicate with others or doesn't have friends, they can fall into a deep chasm. In this place, they are seen and recognized by other people. That social contact is extremely important.

*Being encouraged to start and keep going:* Being part of a creative community not only pays social dividends but it also is very important in starting and sustaining the creative process. Many participants indicate that they found it scary and intimidating in the beginning to join the group and experienced a great resistance to actually starting to be part of a collective creative process. However, they all experienced that the moment you actually join, the community becomes an important motivator and activator to do what you actually want to do. Henry explains that the group has rekindled his enthusiasm for painting and Harold shares that he rarely manages to work on his art at home, but that the environment of the initiative encourages him to actually get things done. He notes that one does not simply sit around doing nothing there.

Facilitators often play an important role in this. They are described as very inviting people, and are often the ones who get participants in the creative mode; later on, they give suggestions on how to develop an artwork, song or scene even further.

Elisabeth: She [the facilitator] is very intuitive and technically very strong. Then she stands behind you and says: “Ooh, that's great, that's going in the right direction.” And then she gives technical advice that you can use. She has a lot of experience. And because I always go so slow and stuff, she says: “this was a huge leap forward!” So sweet.

Christine: He [the facilitator] sometimes comes up with very surprising things. I once made a small statue, I thought of papier-mâché, and then he said, could you make it big too? I've never done that but definitely wanted to try. So then I started doing that, then I made a statue the size of a human being.

Also the others in the group take on that motivating role. Participants emphasize the importance of getting response to your work, working toward a common goal (a performance, play or exhibition) and “being in it together,” which they describe as stimulating. This stems from the principle that when an activity is undertaken collectively, it is much harder for individuals to opt out or neglect their responsibilities to the group. In this way, a sense of commitment and dedication is fostered. The fact that you are all committed to the same thing ensures that you stay focused and work to overcome impediments, whether arising from the mental illness or the artistic process itself, that would be more difficult for you to go through on your own.

Judy: Making music *together* is so important. I need that external stimulus from the group to really let myself go in the music. When I am alone it is so easy to get distracted, for example, by the television.

Hannah: The thing is, when you come here, you can't just give up or say, “Ooh, I'll do it next week.” You really have to go through the problem. You have a commitment with yourself, and also with the people who are there. Because you are not alone, you are part of the group, there is somebody who is directing you and giving you help if you need it.

### Steps toward emancipation

*Stepping out of the patient frame, into an artist frame:* Participants find the experience of participating in an art initiative stimulating and constructive, precisely because there is no therapeutic atmosphere and they are approached as artists and not as patients within this context. The experience of mental illness is not ignored, however, it is neither seen as something negative nor as something that needs to be fixed. Making art is also not seen as an intervention, but as an end in itself.

James: The name “living museum” says it all: when you walk in here you are just an artist. That just helps. And you are also taken seriously. They even say: take yourself seriously as an artist. Seeing yourself as an artist is really taught here … well not taught, but the whole atmosphere is like that.

Patricia: In healthcare, I know that myself, you need a diagnosis to get help at all. That's the entrance, otherwise you won't get it, it won't be reimbursed. It doesn't work like that here. Here you enter and you are addressed as an artist immediately and that is how you are looked at. Even while I'm not yet ready to call myself an artist, that's how I am addressed here because that applies to everyone who comes in here.

All the art initiatives included in this study seem to embody the idea that there doesn't have to be a ready-made solution that solves all problems, but that one should just start doing what one likes and then see where it takes them. This approach, based on positive health and opportunity and an open attitude to what is and what is to come, boosts participants' confidence and clears the way for participants to grow. It makes people aware of their own responsibility and also offers a positive identity that can help them to claim a place in the outside world.

Virginia: Do you know what it is, the approach there is based on the “healthy part,” if you are a little sad or you are stuck, there is time for that, someone takes a moment listening to you and an arm around your shoulder, but then it's like: guys sit down, instruction, let's get to work. And I find that's very good. First you‘re all in your head and [you think] “this is really terrible.” But you get to work and then you think, “Oh, there's more.” And you're having a good time and it's getting a little more normal every time. So that's fantastic. And from that on, you also are an artist in the outside world. “That you say to others: no I can't come because I'm drawing, Or I'm going to drawing class.” And that people immediately say, “Ooh how nice, a lot of fun.” That also has a very … other people like it too.

For many participants participation in a art-initiative is a first step toward a more fully-fledged place in society from a marginalized position. Their participation truly sparked a flow of creativity that gave them a new direction in their lives. Some participants have also been given opportunities by the art initiative to develop further toward professional art practice, starting, for example, with taking on more tasks and responsibilities within the initiative, as a prelude to artistic work outside its walls.

Janet: I'm now an assistant director, supervisor, we do all kinds of exercises and I help, you know? So I've grown a lot there too. [It] has also been quite a process. I'm still an outsider, but now I'm that outsider that makes other people happy, and who isn't bullied but is respected in my differentness.

*Opening the dialogue on psychological suffering:* Finally, a large part of the participants experience that being part of a creative community not only has effects on their own personal lives, but that the effects extend further. Making art, especially in a collective context, as well as its outputs in the form of exhibitions, performance or plays, are a very good medium for communication, and even challenging common public perceptions about mental illness and psychological suffering. For the participants engaged in visual arts and music, this is mostly an implicit process that is related to the fact that the community exists and its output occupies part of the public space. Dialogue about the work arises spontaneously, for example, during exhibitions and performances e.g., when spectators are moved by what is presented, which may lead to conversations about psychological suffering.

Patricia: I don't necessarily find suffering beautiful, but I do think it is good—and this brings us back to the idea of turning your vulnerability into strength—to use it to start a conversation with people, to show that I am not a diagnosis, but a person. Breaking that taboo is really important.

Harold: Emancipation is also part of it. I especially like it when we do an open studio route because then people will come that would otherwise not be in your studio, and you can exchange stories. You make contact with viewers in this case, someone who comes to see the work. And perhaps that will lead to a bit more acceptance.

For the participants involved in theater, it is a much more explicit process. In all theater groups involved in this research, it is common that performances are made about a theme that relates to mental illness or psychological suffering. The aim of these performances is to put difficult experiences in a different light and to open a dialogue. In the process of putting on such a performance the actors exchange a great deal of experiences with each other about the central theme, thus creating a collective experiential knowledge base that forms direct input for the play in the making.

Linda: Our plays are often structured in such a way that the story ends badly, but that leads to a dialogue at the end with the audience. And I honestly think that's the most fun thing to do. It's kind of vulnerable because you're standing in front of the group. We gave a performance for, I believe, 100 people, so there you are. And all kinds of questions are fired at you. But by talking to each other there, they see that reality is very different from the image. In fact, we sometimes ask the question, “Gosh, how would you do that part differently?” And may we invite you to come and play that out? So then one of the actors is taken out and that's where the audience comes in. The actors are then the ones who move along with how the audience tries to give its solution, so to speak.

Participants experience that the moment they enter into a dialogue on their experiences with others *through* their art, different power dynamics apply than when they conduct this kind of conversation in other ways. The artists who invite the viewer(s) to a conversation about the content of the work of art are in charge; after all, they have set the framework for the conversation. It can still feel very vulnerable to do this, participants say, but the context of art makes it a very empowering experience at the same time.

### Opposing forces

*Being inhibited by mental illness:* Participants experience many benefits of making art in their recovery process. However, they all share that it doesn't always work that way. There are times when the threshold to create is so high that they cannot take advantage of the benefits of the creative process. Participants who suffer from depression describe how incipient symptoms can evoke a feeling of inhibition and a sense of worthlessness that prevents them from being creative, and from attending the art initiative. Participants with psychotic experiences emphasize the detrimental effect that psychotic episodes can have on all daily activities, including creative ones, and the adverse effects of anti-psychotic medications on creativity.

Harold: The threshold to get behind a canvas is a bit bigger, then you can feel that it's not worth it or that it's not good enough, or that it's nonsense. Those kinds of ideas can of course bother you and hold you back.

Gary: I feel that my psychosis has an adverse effect on my creativity. It has been very hindering at times, especially due to the use of medication.

All participants emphasize that being part of a creative community helps to keep being creative, or at least be connected with others, even during difficult times. However, such challenges sometimes lead people to stop visiting the art initiative. Maintaining contact with participants during these periods is crucial. Several participants indicate that visiting the initiative is often the first activity they resume after the crisis is over. This is easier when the connection hasn't been broken.

*The experience of too much “flow”:* Just as symptoms of mental illness can hinder creativity, the opposite can also happen. A few of the participants have experienced that through processes in their inner world, they can also end up in *too much* creative flow. This happens especially when they work on something alone. The feeling is often pleasant initially, because then participants can achieve a lot, and they feel sparkling and inspired; however, in the long run the feeling becomes somewhat negative, and actually exhausting. It can even lead to more psychological suffering.

Edward: I think being very creative, being very much in a flow, also has a very bad … something in which you can overshoot. That's something I'm familiar with. That the moment you are in a nice flow, you forget the structure around you and start to freak out a bit and yes, nights go on and in that regard you actually enter a kind of manic period. It's something that's… something to consider. Yes. If at some point I feel like “now it's enough” then I also have to say “now I stop.” As a result, you remain in your own strength and your own peace. Not going into stress mode. But it's such a flow that you can get into. Then it's hard to pull yourself back.

Like Edward, several other participants recognize that it is necessary but sometimes quite difficult to curb creative energy. Frank is often told by people around him that he needs to stop, even when he himself feels that it isn't necessary. “People say, ‘It's not good for you; it's like there's no brake anymore, and you start behaving strangely.” But he feels that in that state, he can set things up much more easily, and everything goes much faster. When he's not in that mode, he actually misses it.

Olivia also struggles with this dilemma: to what extent do you have to slow down creative flow, if it undeniably also has an attractive side?

Olivia: I happen to have a period like this now when I … I just don't really want to stop drawing, practicing, painting, I can get completely exhausted and overstimulated by that. And then I lie in bed at night thinking about everything. After such a period of time I have a period when I just want to do nothing at all for a week and I can't really do anything, because then the dishes are already too much or something. But still. sometimes I want to… If I look at this week, I have gathered so much information and have been really busy with following lessons and trying things with paint and yes when I look at my development then I think: I am developing, that is better than sitting on the couch. But I still have not found the right balance, and I also do not know to what extent that is feasible. Sometimes I just try to accept that this is how it goes.

For some participants, this dilemma is less in the foreground because their art practice mainly takes place in the context of the art initiative, which ensures that there are fairly clear frameworks and boundaries (in time, space and the fact that there are others who know you) that prevent the creative process from becoming excessive.

## Discussion

This study was conducted to gain more insight into the relationship between creativity and personal recovery underlying the claim that art can facilitate health, and in particular, that participatory art activities may facilitate personal recovery from mental illness. Our findings demonstrate that the idea of recovery through creativity resonates with and is rooted in the experiences of the people in this study. In other words, participants indeed find that engaging in creative processes contributes positively to their recovery. The participants' narratives offer insight into the underlying mechanisms of this process, which are situated both in the collective and stimulating aspects of the arts-based intervention, and in the creative and artistic process itself. Their narratives also reveal that people encounter opposing forces, which can make the relationship between creativity and recovery feel like a delicate and precarious balance. These findings can inform the development of activities and policies related to art and recovery in a way that aligns with the needs and experiences of the people concerned.

The findings underscore the important role of the contextual characteristics of arts initiatives in shaping the positive relationship between creativity and recovery. This effect appears to be strongly driven by the emergence of a creative community. Being part of a creative community is a powerful catalyst for all kinds of recovery processes, as has also been demonstrated in previous research (8, 13, 14, 16, 23, 32, 39, 40, 55–57). Key ingredients include “connectedness and acceptance,” “sharing a passion for art,” “not being judged,” “the willingness to help each other,” “commitment instead of pressure,” and “the invitation to be yourself.” These elements create a sense of homecoming, and for many, it means emerging from a situation of social isolation and loneliness and moving toward a situation of (greater) connection and belonging.

Yet, the art initiative offers more than just a place for social connection. It is described as a kind of artistic sanctuary: a space where people reconnect with themselves through creation. It therefore also serves as a space for personal transformation, creative exploration, and identity redefinition. This is linked to the opportunity to step outside the patient frame and into the artist frame, which helps to expand narratives about oneself by developing new, positive identities, while also allowing the “old” identity to remain (and not be rejected by others) [see also: (8, 16, 23, 39, 40, 55, 56, 58)]. In these contexts, art is also naturally used as a means of communication, which can provide participants with opportunities to (re)connect with their communities (13, 39, 59) or even counter stigmas and challenge dominant ways of conceptualizing mental illness (56).

The fact that the positive relationship between creativity and recovery is so dependent on the context of the art initiative, as shown in our and other studies, raises the question of whether creativity and recovery *per se* have anything to do with each other. After all, mechanisms such as the development of positive identities and empowerment through meaningful activity, group interaction and connection with a community are very similar to findings from studies on recovery programs in a broader sense [see for example the work on Recovery Colleges: (60–62)], which may indicate that it is primarily the setting itself and engagement with a meaningful topic or activity—of any kind—that is associated with recovery. However, that would not do full justice to the stories of our participants, which seem to underline that creativity and making art *in themselves* have unique characteristics that can change the experience of mental illness and promote recovery, although associated mechanisms remain relatively underexplored in the existing literature.

In line with previous literature. (8, 13, 16, 23, 32, 39, 40, 55, 57, 63), our research confirmed that within the flow and timelessness of creative processes, negative thoughts and emotions fade into the background, and creativity can improve mood and generate a deep sense of satisfaction. The creative process, therefore, also functions as a coping mechanism for participants—one that is experienced as healthier than the (usually more destructive) coping strategies they may otherwise be familiar with. Moreover, the creative artistic process offers the opportunity to express, make tangible, transform, and process difficult and painful experiences. This, too, is a form of coping, giving meaning and space to one's own experiences.

In our study, however, we also saw a much deeper meaning of creativity. We saw how important creativity and art-*making* are to our participants in understanding themselves and the world around them. Being creative and being drawn to art is part of who they are, or, as Stacey & Stickley (59) put it, creativity represents an integral aspect of the person's perception of themselves, as for many it is an essential component of the way they wish to live their life. For them, recovery from mental illness therefore naturally includes recovering their creativity as a central feature of their identity. This entails not only restoring creativity but also (re-)engaging in aesthetic experiences, particularly experiences of “beauty” and “wholeness”, as these kind of experiences appear to have potential for eudaimonic wellbeing and transcending painful and alienating experiences on the road to recovery. In fields such as creative therapy (64), public art (65), care ethics (66), and neuroaesthetics (67), the importance of aesthetic experiences in relation to wellbeing, psychological healing, and health has already been emphasized. However, this topic has remained underexplored within research on recovery and recovery-supporting practices. Nevertheless, it is not far-fetched to connect concepts of recovery and aesthetics, particularly within a hermeneutic framework.

According to Gadamer (68), a leading figure in modern hermeneutic philosophy, our main source of suffering as humans is our incompleteness. Following Gadamer's vision of aesthetics, “beauty” is a way of overcoming incompleteness, because it represents an “earthly truth” about who and how we (essentially) are. In the moment of the experience of “the beautiful,” we recognize our (full) selves in a sense, and this experience can provide relief and make us feel like a complete person, even if only for a moment. The participants‘ stories in our study seem to resonate with this idea: the effect art-making has on the self-image, self-acceptance and the sense of meaningfulness and connectedness has common ground with the idea of overcoming incompleteness and returning to “being human.” From Gadamer, we can hypothesize that art may provide a unique access to a sense of completeness or “wholeness” that is difficult to access through other avenues, and which could explain the specific effect of art on people recovering from mental illness [see also: (21, 58, 69)].

Evidently, this constitutes a domain that warrants further scholarly inquiry, ideally through a multidisciplinary approach. Such investigation may contribute to a reconsideration—and potential repositioning—of the concept of ‘suffering' within the recovery paradigm. Within recovery thinking, people with mental illness are invited to confront their suffering, to name it as part of their life story, and to move forward to a position where they are no longer defined by it. Considering suffering as an inherent condition of human existence, just like beauty, where both extremes can even merge in the experience of art, offers an alternative or broader perspective on this idea, which may be relevant as a basis for sustainable recovery.

Other avenues for further research raised by this study include the differences between art disciplines, whereby each discipline may have its own specific opportunities in the recovery process, as well as drawing attention to the opposing forces that stand in the way of recovery through art. With regard to this last point, some authors have pointed to challenges related to art-making, including encountering frustration and experiencing the gap between wanting and being able (70); the possibility of triggering negative, distressing memories (13); struggles with self-esteem and motivation (71); and feelings of “emptiness” once workshops end (38). To our knowledge, however, the specific challenges found in our study (inhibition and acceleration of the creative process) have not yet been extensively studied in relation to recovery. Exploring these opposing forces has relevance to practices that focus on recovery from mental illness through art and face challenges when participants do not seem to benefit from the art environment or even drop out.

### Strengths and limitations

While the strengths of our study include the large sample size and the breadth of the findings due to the inclusion of participants from different art disciplines and organizations, the study also has several limitations. While the strength of the interpretive methodology, based on experience, lies in its ability to shed light on the meanings of various aspects of creativity and recovery, a drawback of the methodology is the difficulty of distinguishing between recovery *mechanisms* and *outcomes*. Because the pursuit of a robust, data-driven theory—as is common in, for example, a grounded theory design—was not the goal, the presented results remain rather noncommittal and exploratory in nature, leaving considerable room for interpretation and many unanswered questions. Other limitations include the absence of a member check, uncertainties about the nature and seriousness of the participants' psychological complaints (because these were not measured), and the limitedness of a single interview-methodology to capture the dynamic nature of the recovery process. In retrospect, the difference between different art disciplines (visual vs. performing) should perhaps have taken a more central place in the main question and design of the study, as well as the difference between people who were already fascinated by their art discipline as a child, and people who only came into contact with their art discipline after the onset of mental illness.

Despite the limitations, we believe the study contributes to the theoretical understanding of recovery through art in a way that does justice to and promotes the voices of people with lived experiences, with relevance for mental health care and art policy.

### Practical implications

The results confirm the importance of the existence of accessible creative sanctuaries and communities where people can make art and work on their recovery. Furthermore, it is important that caregivers of people with a creative affinity understand the meaning of creativity and consider the necessity of recovering creativity during times of relapse and crisis, as restoring creativity can potentially act as a catalyst for recovery in all other areas.

## Data Availability

The datasets presented in this article are not readily available because the dataset consists of rich and complex qualitative data that is difficult to anonymize without losing crucial information. Data can be requested from the lead author, but any portions that may contain identifiable information will be removed. Requests to access the datasets should be directed to Jenny Boumans, Jboumans@trimbos.nl.
